# Assessment of Oral Mucosal Lesions (OML), Periodontal Health Conditions, and Unmet Dental Treatment Needs in the Rural Adult Population of Jharkhand, North India

**DOI:** 10.7759/cureus.49961

**Published:** 2023-12-05

**Authors:** Sandeep Kumar, Tapan K Mandal, Arunoday Kumar, Manawar Ahmad, Hina Naim, Jagadesaan N

**Affiliations:** 1 Public Health Dentistry, Rajendra Institute of Medical Sciences, Ranchi, IND; 2 Conservative Dentistry and Endodontics, Rajendra Institute of Medical Sciences, Ranchi, IND; 3 Prosthodontics and Crown & Bridge, Regional Institute of Medical Sciences, Imphal, IND; 4 Prosthetic Dental Sciences, College of Dentistry, Jazan University, Jazan, SAU; 5 Prosthodontics and Crown & Bridge, JKK Nattraja Dental College and Hospital, Komarapalayam, IND

**Keywords:** clinical attachment loss (cal), unmet dental treatment needs, periodontal diseases, community periodontal index (cpi), oral mucosal lesions (oml)

## Abstract

Background

Dental diseases like caries, periodontal diseases, and oral mucosal lesions (OML) are common findings in rural adult populations that greatly impact their quality of life.

Aim

To assess OML, periodontal health conditions, and unmet dental treatment needs in the rural adult population in Jharkhand.

Methodology

A total of 700 permanent residents of Bero Block, Jharkhand, North India, in the age group of 35-44 years, participated in this cross-sectional study. Both men and women were equally represented. Their socio-demographic characteristics and previous dental visits were collected using standardized proforma. An assessment of periodontal health conditions, OML, and unmet dental treatment needs was done using the World Health Organization (WHO) Oral Health Assessment Proforma of 1997.

Results

It was found that over half (54.3%) of the study population had the adverse habit of smoking and chewing *paan*. Males were more likely to experience leukoplakia (18.87%), whereas females were more likely to experience abscesses (9.43%). The majority of males and females had a community periodontal index (CPI) score and loss of attachment (LOA) score greater than two, which indicated poor periodontal health as assessed by the periodontal index. Both males and females needed extraction of the diseased teeth as their primary treatment.

Conclusion

The rural adult population residing in the Bero block of Jharkhand showed poor periodontal health and high unmet dental treatment needs. These people need effective oral health promotion policies and dental health education to improve their oral health.

## Introduction

Dental health is an important and integral part of contributing to the overall health of an individual, which goes beyond just having healthy teeth. The factors that are essential for having good oral health include healthy gums, proper oral hygiene practices, periodic dental checkups, a balanced diet, prevention of tobacco and alcohol consumption, stress management, etc. [1]. To have optimal oral health, these factors have to be taken care of as needed. Oral diseases are extremely prevalent and have a significant social impact, making them a health concern [2]. There are several systemic diseases associated with dental caries like infective endocarditis, cardiovascular diseases, diabetes mellitus, bacterial pneumonia, chronic obstructive pulmonary disease, and gum disease that impact 60% to 80% of the Indian population and almost half of the world’s population, that is, 45% or 3.5 billion people globally [3].

Oral mucosal lesions (OML), periodontal diseases, and unmet dental treatment are often subjects of concern. OML are generally the abnormal tissue lining of the oral cavity due to a wide variety of reasons like trauma, chronic oral inflammatory diseases, oncogenic processes, etc. Additionally, tobacco use is a major contributor to the development of oral mucosal lesions. There is a wide spectrum of oral mucosal alterations and lesions that can result from tobacco use, whether it is smoked or smokeless. A tobacco user's type, manner, and frequency of usage will determine the type and location of the lesion. These lesions may appear in different sizes and structures and occur at different places. These lesions may be leukoplakia, malignant tumors, lichen planus, candidiasis, ulceration, abscess, etc. OML are usually associated with various local and/or systemic conditions and have been reported in 41.2% of the Indian population [4]. The patient's eating, drinking, and speaking habits are affected by OML; as a result, the person's regular activities are hampered because of the pain and discomfort caused by these lesions [5].

Periodontal diseases are very common in the rural adult population. Several factors may contribute to this, including a low level of awareness about oral health, financial constraints, and a lack of oral health care facilities [6]. Periodontal disease manifests itself in the form of bleeding from gums, gingival recession, food lodgement, interdental spacings, halitosis, and others [7].

Unmet dental treatment needs are in those cases that need dental treatment but do not get a dental care assessment in the required timeframe [8].

There have been minimal studies carried out in Jharkhand, especially in the rural population, wherein their oral health and unmet dental needs have been assessed. Hence, this study was carried out to assess OML, periodontal health conditions, and unmet dental treatment needs in the rural adult population in Jharkhand.

## Materials and methods

This cross-sectional study was conducted in Bero block, Jharkhand, and included adults in the age group of 35-44 years. The study took place between January 2022 and May 2023 (14 months). Based upon the findings of the pilot study and using the formula recommended by the World Health Organization (WHO) for sample size calculation [9], it was found that a minimum number of 374 would be sufficient. As per the recommendations of the WHO, the formula of N=z2p(1-p)/d2 was used for calculating the sample size where z is the statistic corresponding to the level of confidence, p is the expected prevalence and d is the precision value. Keeping the z value to be 1.96, prevalence to be 42%, and precision value to be 0.05 in the above formula, a minimum sample size of 374 was obtained. We decided to include 350 males and 350 females after doing the necessary design effect corrections for cross-sectional studies.

These studies included all adults (over 35 years of age) who were willing to participate and were residents of Bero block for at least 10 years. The study excluded participants who were unwilling or medically compromised. To select the study population, a simple random sampling method was used.

The study was conducted after obtaining approval from the Institutional Ethics Committee at Rajendra Institute Of Medical Sciences (RIMS), Ranchi, with institutional ethical clearance number IEC RIMS Memo No. 282. The socio-demographic data, periodontal health conditions, OML, and unmet dental treatment needs were assessed according to the WHO Oral Health Survey assessment forms. To assess periodontal health, the community periodontal index (CPI) and loss of attachment (LOA) index were used as per the WHO recommendation [9]. As per the WHO CPI, the status of the periodontium of 10 teeth was examined and evaluated with a 0.5 mm ball tip, with the WHO CPI probe present in the oral cavity. The scoring was done depending on the clinical finding associated with the individual teeth, as per the literature available [9]. The teeth were assessed clinically and the CPI score was assigned in the form of a numeric score ranging from 0 to 4. CPI scores 0, 1, 2,3, and 4 represent healthy periodontium, bleeding of the gingiva, calculus, and bleeding of the gingiva, a periodontal pocket of 4-5 mm, and a periodontal pocket of ≥ 6 mm, respectively. The teeth present in the oral cavity were also examined and assessed for LOA. The teeth were assessed clinically and the LOA score was assigned in the form of a numeric score varying from 0 to 4. The LOA scores of 0, 1, 2, 3, and 4 show a loss of attachment of about 0-3 mm, 4-5 mm, 6-8 mm, 9-11 mm, and ≥12 mm respectively [9]. 

The data were collected through various dental camps organized in Bero block, Jharkhand. As part of the data collection process, the dentist was trained on how to perform various oral exams based on standardized criteria. A mouth mirror, a CPI probe, and a light source were used in the oral examination.

The study instruments were sterilized after use. In the event of an emergency oral health condition requiring immediate intervention, the patient was referred to a trained specialist right away.

An analysis of the data was conducted using SPSS (IBM Corp. Released 2011. IBM SPSS Statistics for Windows, Version 20.0. Armonk, NY: IBM Corp). The data were distributed based on their frequency. Chi-square tests were performed on categorical variables. When a p-value of 0.05 was used, a statistically significant difference was considered.

## Results

More than two-thirds of the study population were literate, working, and did not suffer from any systemic diseases. More than half (54.3%) of the study population had the adverse habit of tobacco and *paan* consumption. The majority (92.6%) of them had never visited a dentist before. Table 1 shows the sociodemographic characteristics of the study population based on several factors like age, education, occupation, habits, etc.

**Table 1:** Sociodemographic characteristics N(%): number of participants in the study.

Factor	Categories	N (%)
Mean age	Male	40.0±2.0
	Female	39.0±2.0
Gender	Male	350 (50.0%)
	Female	350 (50.0%)
Education	Literate	490 (70.0%)
	Illiterate	210 (30.0%)
Occupation	Working	483 (69.0%)
	Not working	217 (31.0%)
Adverse habit	Yes	380 (54.3%)
	No	320 (45.7%)
Systemic disease/Long-term medication	Yes	212 (30.2%)
	No	488 (69.8%)
Previous dental visit	Yes	52 (7.4%)
	No	648 (92.6%)

Males and females had significantly different OML as shown in Table 2.

**Table 2:** Oral mucosa condition of males and females p-value: level of significance.

Condition	Male	Female	Chi-Square test (p-value)
No abnormal condition	184 (52.57%)	257 (73.42%)	<0.0001**
Malignant tumor	0 (0.00%)	0 (0.00%)	
Leukoplakia	66 (18.87%)	12 (3.43%)	
Lichen planus	45 (12.87%)	15 (4.29%)	
Ulceration	13 (3.71%)	17 (4.86%)	
Candidiasis	18 (5.1%)	11 (3.14%)	
Abscess	16 (4.58%)	33 (9.43%)	
Other conditions	8 (2.30%)	5 (1.43%)	

In males, leukoplakia (18.87%) was the most common lesion found followed by lichen planus (12.87%). In females, abscesses (9.43%) followed by ulceration (4.86%) were more commonly observed.

In males, 61 (17.4%) showed the homogenous form of leukoplakia and 5 (1.4%) showed a speckled type. Among females, 11 (3.14%) females showed a homogenous form and 1 (0.2%) showed the speckled type of leukoplakia. More than two-thirds of the males (88.8%) and females (80.0%) showed the reticular form of lichen planus. The plaque form was observed in five (1.42%) males and three (0.85%) females. The aphthous ulcer was observed in 8 males (2.3%) and 11 females (3.14%). A traumatic ulcer was diagnosed in four males (1.1%) and five females (1.4%). A herpetic ulcer was observed in one male (0.3%) and one female (0.3%).

The majority of males showed a periapical abscess (2.9%), followed by periodontal (1.1%) and gingival abscesses (0.6%). In females too, the majority showed a periapical abscess (5.7%), followed by periodontal (2.3%) and gingival abscesses (1.4%).

The periodontal health condition was assessed by the CPI and LOA scores and the findings are tabulated in Table 3.

**Table 3:** Periodontal health condition assessed by the CPI and LOA scores p-value: level of significance, CPI: community periodontal index, LOA: loss of attachment

Factor	Categories	Male	Female	Chi-square test (p-value)
CPI Score	Score 0	10 (2.89%)	30 (8.57%)	<0.0001**
	Score 1	25 (7.14%)	56 (16.00%)	
	Score 2	45 (12.86%)	88 (25.14%)	
	Score 3	85 (24.29%)	128 (36.58%)	
	Score 4	185 (52.82%)	48 (13.71%)	
LOA Score	Score 0	12 (3.43%)	28 (8.00%)	<0.0001*
	Score 1	28 (8.00%)	58 (16.57%)	
	Score 2	54 (15.43%)	92 (26.29%)	
	Score 3	66 (18.86%)	122 (34.85%)	
	Score 4	190 (54.28%)	50 (14.29%)	

The results of Table 3 show that males and females had significantly different periodontal health. More than half of the males showed a CPI score of 4 (52.82%) and an LOA score of 4 (54.28%). The majority of the females showed CPI and LOA scores of 2 or 3.

Depending on the research study, it was found that different kinds of dental treatment were needed for males and females, which is illustrated in Table 4.

**Table 4:** Treatment needs

Need assessment	Male	Female
Preventive care	0 (0.00%)	0 (0.00%)
Fissure sealant	0 (0.00%)	0 (0.00%)
One surface filling	76 (21.71%)	82(23.43%)
Two surface filling	88 (25.14%)	77 (22.00%)
Crown for any reasons	33 (9.43%)	11 (3.14%)
Veneer and Laminates	11 (3.14%)	18 (5.14%)
Pulp care and restorations	136 (38.86%)	88 (25.14%)
Extractions	250 (71.43%)	212 (60.57%)
Need for other care	11 (3.14%)	13 (3.71%)

Extraction, followed by pulp care, was the major treatment needed in both males and females. In addition, one and two surface fillings were also required in most of the males and females. Those study population who were diagnosed with different oral mucosal lesions (OML) and periodontal diseases were referred to the Department of Oral Medicine and Radiology and the Department of Periodontology, respectively, at RIMS, Ranchi, Jharkhand, for the needful treatment.

## Discussion

This study was carried out to assess OML, periodontal health conditions, and unmet dental treatment needs in the rural adult population in Jharkhand. Every attempt was taken to match the age and gender of study participants to avoid bias. More than half of the study population exhibited the adverse habit of chewing tobacco and gutkha, indicating that people were not aware of the harmful effects of tobacco. Tobacco contains a harmful chemical called nicotine, which has adverse effects on oral health [10,11]. The adverse effects include the development of periodontitis, halitosis, development of oral mucosal lesions, oral cancer, etc. Several Indian studies conducted on rural populations have demonstrated that tobacco is consumed by the majority of people residing in rural areas [12,13].

Most of those surveyed have never been to a dentist. This was consistent with the findings of the studies carried out by Kumar et al. [14] and Okunseri et al. [15]. There is a lack of awareness about periodic dental checkups by the Indian masses and therefore, people only visit the dentist when there is an urgent need or when the pain does not subside by home-applied remedies.

More than half the study population reported lesions associated with tobacco consumption. Approximately 37% of males and females reported two forms of OML, i.e., leukoplakia (homogeneous or speckled type) and lichen planus (reticular or plaque form), and nearly 20% of total males and females had abscesses (periapical, periodontal, or gingival abscess) and ulceration (aphthous, traumatic, or herpetic ulcer), which are associated with tobacco consumption and poor oral hygiene, as reported by several Indian studies [16,17]. The authors believe that the concept of preventive care needs to be instilled in the mindset of the Indian population, as the rural populations have poor oral health awareness and adhere to traditional methods of oral hygiene practices, which may not be effective every time and may create serious life-threatening problems. Therefore, there is an urgent need to create oral health awareness and needful action for the “oral health promotion” of the rural population.

The majority of the males and females who were included in the study group showed CPI and LOA scores of more than two. This was suggestive of the fact that they had poor periodontal health. The noticeable signs of gingival bleeding, gingival recession, gingival abscess, periodontal abscess, periodontal pocket, food lodgement, and halitosis are common markers of poor periodontal condition. Several studies conducted on the Indian population residing in rural areas have shown that they have poor periodontal health [18,19]. The authors believe that besides the aging process, lack of oral health awareness, poor utilization of dental services, and improper oral hygiene practices may be possible causes for poor periodontal health in the study population. The diagnosis of gingival and periodontal diseases was based upon the codes and criteria recommended by the CPI-LOA index of WHO Oral Health Assessment Performa 1997.

In the study population, extraction was found to be a major treatment need in both males and females. There was a lack of utilization of dental services as reported in our study. The concept of preventive care was missing in the study population. Early intervention in the development of dental diseases could have prevented the teeth from being extracted at earlier stages. Hence, effective policies for the upliftment of oral health should be drafted for this study population.

## Conclusions

Good oral health condition plays a vital role in a person’s life. Hence, timely consultation with dentists regarding problems with one's teeth is vital. In the present study, it was found that a majority of the participants had never visited a dentist, had poor periodontal health, habitually consumed tobacco and gutkha, and had unmet dental care. Furthermore, a majority of the male and female patients needed both dental extraction as a major treatment and oral health awareness.
